# A Multimodal Biomicroscopic System based on High-frequency Acoustic Radiation Force Impulse and Multispectral Imaging Techniques for Tumor Characterization *Ex vivo*

**DOI:** 10.1038/s41598-017-17367-1

**Published:** 2017-12-13

**Authors:** Jihun Kim, Anna Seo, Jun-Young Kim, Sung Hyouk Choi, Hyung-Jin Yoon, Eunjoo Kim, Jae Youn Hwang

**Affiliations:** 1 Daegu Gyeongbuk Institute of Science & Technology, Department of Information & Communication Engineering, Daegu, 42988 Republic of Korea; 2 Kyungpook National University, 3D Convergence Technology Center, Daegu, 41061 Republic of Korea; 3 Kyungpook National University Hospital, Department of Orthopedic Surgery, Daegu, 41944 Republic of Korea; 4 Seoul National University, College of Medicine, Seoul, 03080 Republic of Korea; 5 Daegu Gyeongbuk Institute of Science & Technology, Department of Nano & Energy Research, Daegu, 42988 Republic of Korea

## Abstract

We report a multimodal biomicroscopic system which offers high-frequency ultrasound B-mode, acoustic radiation force impulse (ARFI), and multispectral imaging for qualitative tumor characterization *ex vivo*. Examinations of resected tissues from diseased regions such as tumors are crucial procedures during surgical operations to treat cancer. Particularly, if tiny tumors remain at surgical sites after tumor resection, such tumors can result in unwanted outcomes, such as cancer recurrence or metastasis to other organs. To avoid this, accurate characterizations of tumors resected during surgery are necessary. To this end, we devised a multimodal biomicroscopic system including high-frequency ultrasound B-mode, ARFI, and multispectral imaging modalities to examine resected tumors with high levels of accuracy. This system was evaluated with tissue-mimicking phantoms with different mechanical properties. In addition, colorectal tumors excised from cancer patients were examined. The proposed system offers highly resolved anatomical, mechanical, chemical information pertaining to tumors, thus allowing the detection of tumor regions from the surface to deep inside tissues. These results therefore suggest that the multimodal biomicroscopic system has the potential to undertake qualitative characterizations of excised tumors *ex vivo*.

## Introduction

Cancer is the second most common cause of death in the United States as well as a major public health problem worldwide^1^. Despite significant technological advancements related to the diagnosis and treatment of cancer, only a limited improvement in the overall survival rate has been realized due to the heterogeneous patterns of cancer^2^. In cancer patients, one of the devastating events is the growth of secondary primary tumors or distant cancer metastasis after a surgical operation to treat cancer. In particular, if tiny residual tumors are not detected during surgical operations and therefore remain at the surgical site, they can result in the recurrence of cancer or in metastasis to other organs^3–6^. Therefore, the precise and rapid tumor detection of excised tumors has become one of the most important tasks during surgical operations for cancer, as it allows confirmation of whether tumors remain at the surgical site after tumor resection^7^. However, to determine whether the excised tissues are properly acquired and furthermore whether the additional dissection of tissues is required during surgery, the excised tumors are transferred to pathologists, who examine the specimens under conventional microscopes and make decisions by investigating the deviations in the cell morphologies and changes in the cell distributions across the tissue samples. However, this process is time-consuming, inconsistent, and subjective as inter- and intraobserver variations^8^. Therefore, an advanced method capable of overcoming this shortcoming would be beneficial for the rapid characterizations of excised tumors during surgery.

Currently, a number of techniques, including spectral^9^ and ultrasound imaging^10^, have been developed for the characterization of excised tumors. Among them, spectral imaging is highly useful as a non-invasive imaging modality for the characterization and delineation of tumors in excised tissues. For this reason, it has been explored for various clinical and biological applications^11–13^. In particular, it was utilized to discriminate between pre-cancerous/cancerous tumors and normal tissues both *in vivo* and *ex vivo* ^14^. As a result, malignant tumors were successfully distinguished from normal tissues^15^. In that study, different auto-fluorescence emission spectra were exhibited. Moreover, adenomatous and normal colonic tissues could be clearly discriminated via the spectral imaging of the colorectal regions of interest when they were excited by ultraviolet light^16^. As demonstrated by these earlier works, spectral imaging has shown great promise for discriminating between normal tissues and tumors. However, the use of the spectral imaging and analysis during tumor detection in tissues is limited to imaged tissues with shallow penetration depths, as light cannot penetrate deep inside tissues. Therefore, other advanced imaging techniques must be applied to detect tumor regions of interest, which require further resection during surgical operations deep inside the tissues.

Ultrasound imaging modalities such as ultrasound B-mode imaging and acoustic radiation force impulse (ARFI) imaging may complement the limited capability of spectral imaging for the detection of tumors deep inside excised tissue due to the permeability of ultrasound waves in tissues. The high-frequency ultrasound B-mode imaging offers structural information about the organs or tissues of interest in more detail than conventional B-mode imaging because the high-frequency ultrasound improves both the axial and lateral resolution by sacrificing the penetration depth^17,18^. The −6 dB lateral beam width is inversely proportional to the ultrasound frequency^19^. Therefore, high-frequency ultrasound imaging techniques have been applied to characterize excised tissues and to observe the subsurface of living tissues, which typically cannot be examined when using an optical microscopic system owing to the limited penetration depth of light noninvasively at resolutions similar to that of an optical microscopic system^20^. Using high-frequency ultrasound imaging, various malignant lesions *in vivo* and *ex vivo* have been detected^21–23^. However, small and dense tissues may cause false-negative results during attempts to detect tumors using B-mode imaging because both dense breast and tumor regions exhibit similar acoustic properties^24–26^. Thus, an additional reliable method may be needed to detect tumors more accurately. High-frequency ARFI imaging or shear wave elasticity imaging (SWEI) have been shown to be a viable alternative to overcome the shortcomings of B-mode imaging by overlaying a map of the stiffness of tissues with an ultrasound B-mode image^27,28^. It uses high-intensity ultrasound pulses with short durations to apply acoustic radiation force to a target, which results in displacements of the target tissues, where the displacements depend on their mechanical properties. By quantification of the displacements of tissues at a high resolution, the mechanical properties of the target can therefore be measured. Discrimination between soft tissues and tumors, not always possible with B-mode imaging, can thus be realized by high-frequency ARFI imaging. However, high-frequency ultrasound B-mode and ARFI imaging typically offers two-dimensional morphological and mechanical information about a target tissue in the axial direction instead of molecular information on the surface of the tissue in the transverse direction, as in multispectral imaging.

All of the high-frequency ultrasound and optical imaging technologies mentioned above have inherent capabilities in offering specific information. However, when high-frequency ultrasound B-mode, ARFI, and multispectral imaging methods are used in isolation for the characterization of tumors excised during surgery, they may show limited capabilities, such as the low penetration depth of spectral imaging, the low image contrast of B-mode imaging, and the lack of anatomical information by ARFI imaging, resulting in low detection accuracy in such examinations. On the other hand, a combination of multiple imaging modalities may be beneficial to detect and characterize diseased tissues since their combination can offer complementary and even synergetic information regarding the detection and characterization of diseased tissues^29^. For these reasons, a detailed, highly accurate, and rapid examination of the distribution and invasion depth of tumors in excised tissues during surgery may benefit from the use of multiple imaging modalities simultaneously, including high-frequency ultrasound B-mode, ARFI and multispectral imaging, as such a combination can allow one to obtain multiple/complementary information about excised tumors from the surface to areas deep inside tissues, such as the surface margin, anatomy, and mechanical properties of tumors. Note that mechanical, anatomical, and molecular information has been shown to be crucial in the detection of tumors *ex vivo*. Tumors have typically been shown to be denser and stiffer than normal tissues, and tumors exhibit different spectral signatures compared to normal tissues due to their different molecular characteristics.

In this paper, we therefore demonstrate a novel multimodal biomicroscopic system for rapid examinations of excised tumors with high levels of accuracy. The system combines tri-imaging modalities: (1) high-frequency ultrasound B-mode imaging, which is capable of an examination of the anatomy of tissues in the longitudinal direction; (2) high-frequency ARFI imaging, which allows measurements of the mechanical properties of the tissues qualitatively; and (3) multispectral imaging, which enables one to discriminate chemical properties on the surfaces of tissues. For an evaluation of the multimodal biomicroscopic system, a total of nine tissue-mimicking phantoms with different mechanical properties were examined. We also investigated whether the multimodal biomicroscopic system allowed us to differentiate colorectal tumors from normal regions in tumor regions resected from six cancer patients, thereby demonstrating the potential of the multimodal biomicroscopic system for tumor characterizations *ex vivo*, which may be very useful for tumor characterizations during surgery.

## Results

### Tissue-mimicking phantom study for an evaluation of the multimodal biomicroscopic system

**Figure 1:**
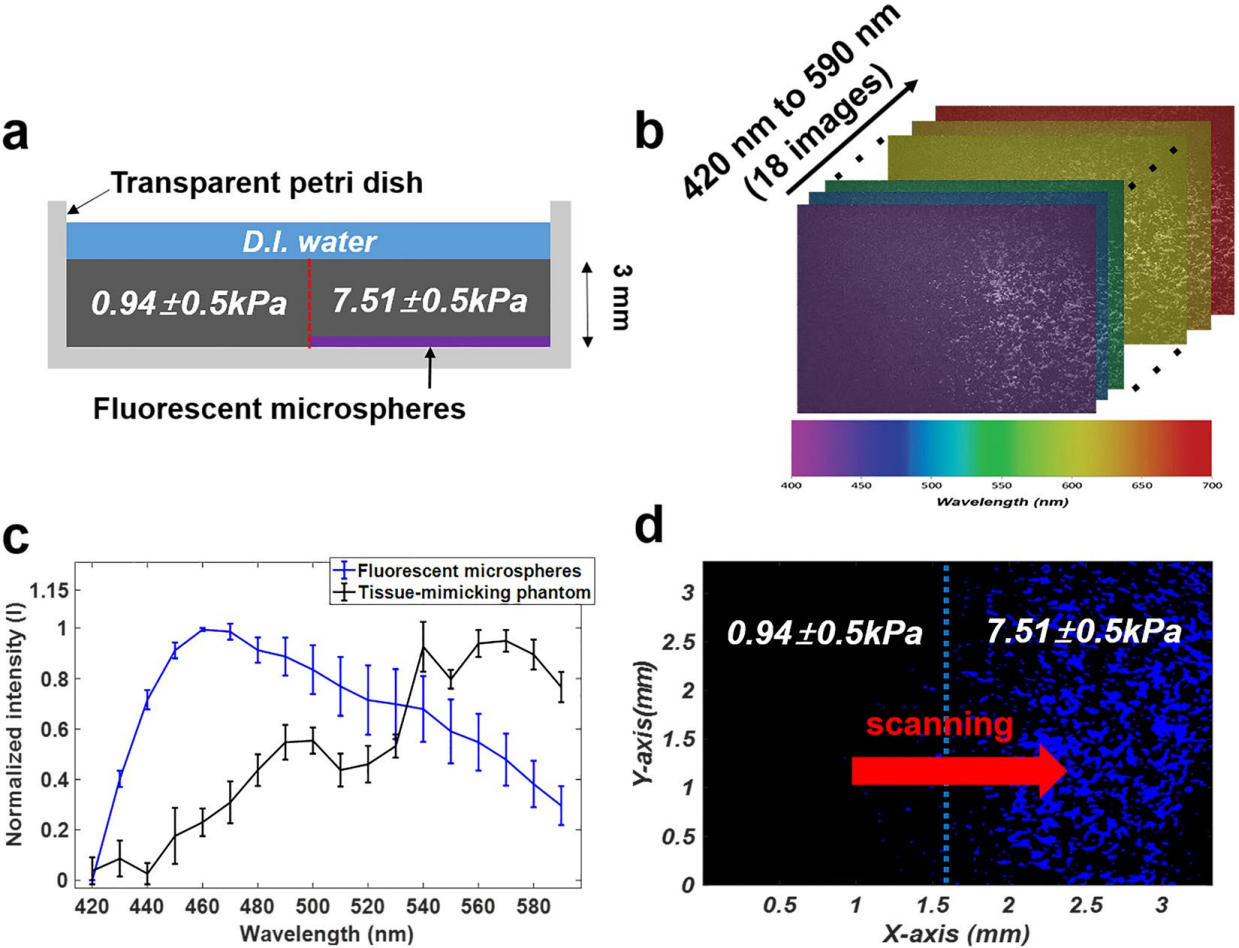
Evaluation of the multispectral imaging system with a tissue-mimicking phantom. (**a**) Structure of the bilayer tissue-mimicking phantom in lateral direction. (**b**) Fluorescence spectral image cube. (**c**) Normalized spectral signatures of the fluorescent microspheres and gelatin phantom. (n = 10) (**d**) Spectral classified image of the gelatin phantom.

For an evaluation of the multimodal biomicroscopic system, we carried out multimodal imaging of a total of nine tissue-mimicking phantoms with different mechanical properties. Figure 1a shows the internal structures of the bilayer tissue-mimicking phantom in the lateral direction. Multispectral imaging of the tissue-mimicking phantom was initially performed to obtain fluorescence spectral images within the wavelength range of 420 nm to 590 nm with a step size and a bandwidth of 10 nm (Fig. 1b). The normalized spectral signatures of the fluorescent microspheres and the gelatin phantom were clearly discernable, as shown in Fig. 1c. The spectral signature of the fluorescent microspheres showed a peak intensity level at ~460 nm, whereas the peak intensity of the spectral signature of the gelatin phantom was found to be ~570 nm. With these normalized reference spectral signatures, the spectral classified image was obtained (Fig. 1d). In the image, the blue-colored regions represent the fluorescent microspheres, whereas the black-colored regions represent the gelatin phantom. Here, the stiffer regions, which included the fluorescent microspheres, were clearly distinguished from the tissue-mimicking phantom on the surface of the phantom by the multispectral imaging and analysis.

**Figure 2:**
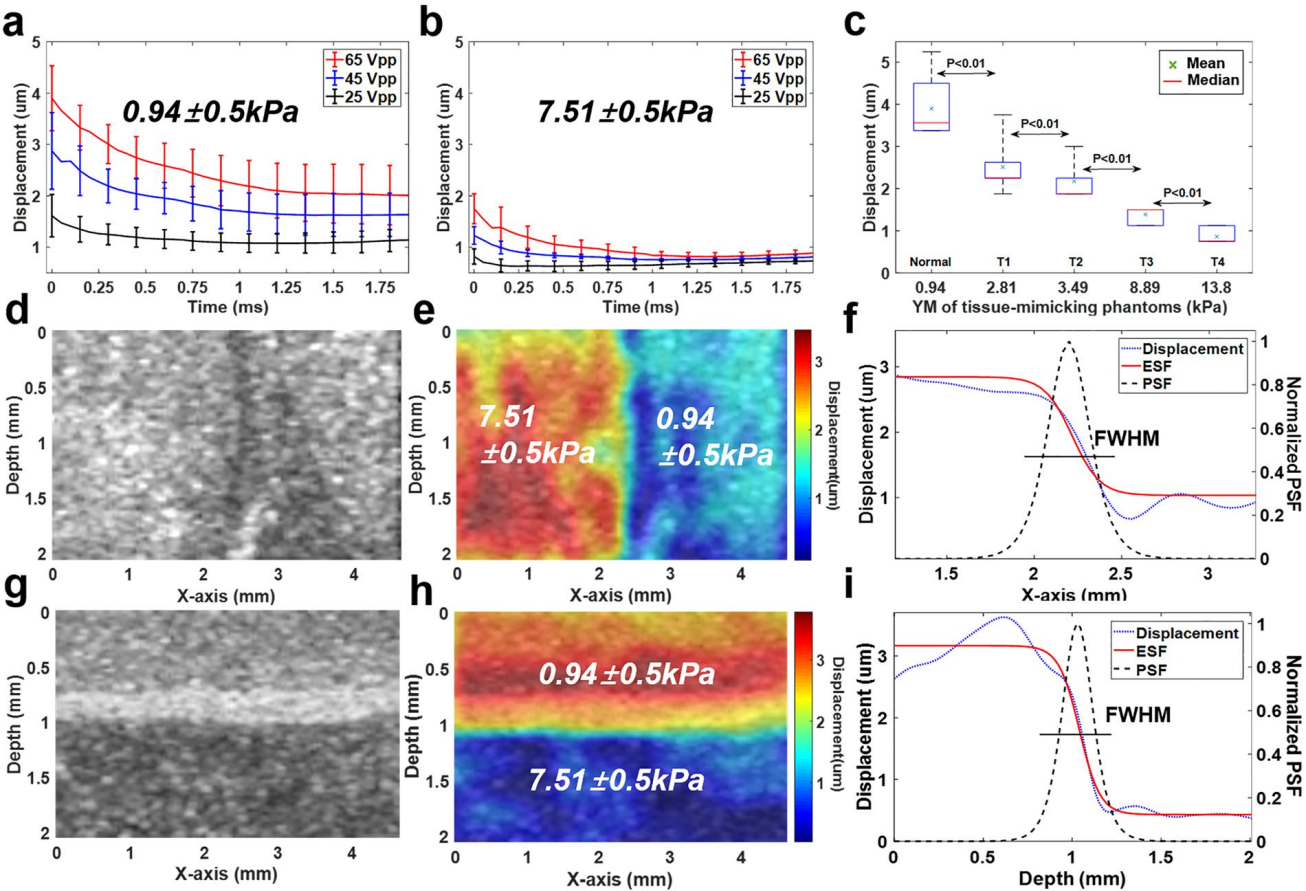
Evaluation of the ultrasound biomicroscopic system with gelatin phantoms. (**a**,**b**) Graphs of the mean displacements of the gelatin phantoms with YM of 0.94 ± 0.5 kPa and 7.51 ± 0.5 kPa under each excitation condition (n = 10). (**c**) Box and whisker plot of the displacements of gelatin phantoms at different YM corresponding to the T-stages of cancer (n = 10). The red lines indicate the median values whereas the marks, ‘x’ indicate the mean values (**d**,**e**) High-frequency B-mode and ARFI images of bilayer gelatin phantoms in the lateral direction. (**f**) Lateral resolution measured in a point-spread function constructed at the boundary between two layers. (**g**,**h**) High-frequency B-mode and ARFI images of bilayer gelatin phantoms in the axial direction. (**i**) Axial resolution measured in a point-spread function constructed at the boundary between two layers.

B-mode and ARFI imaging of the tissue-mimicking phantom were also performed using the ultrasound microscopic system. Before performing B-mode and ARFI imaging, to identify the excitation parameters of the acoustic radiation force of the transducer, which is capable of inducing proper displacements in tissue-mimicking phantoms with two different Young’s modulus (YM) corresponding to tumors and normal tissues, the dynamic displacements of particles on the surface in the tissue-mimicking phantom were measured within the focal region of the transducer at each excitation condition. The pulse duration of 45 MHz sinusoidal bursts was 0.22 ms (number of cycles: 10,000). The negative acoustic pressures for ARFI imaging were 1.37, 1.65, and 1.95 MPa. The means of the maximum displacements for regions with a YM value of 0.94 kPa were measured to be 1.61 ± 0.41, 2.86 ± 0.75, and 3.9 ± 0.63 μm at acoustic pressures of 1.37, 1.65, and 1.95 MPa, respectively (Fig. 2a), whereas the means of the maximum displacements for regions with a YM value of 7.51 kPa were measured to be 0.82 ± 0.15, 1.23 ± 0.17, and 1.75 ± 0.29 μm at acoustic pressures of 1.37, 1.65, and 1.95 MPa (Fig. 2b), respectively. In the figures, the smaller displacements represent the stiffer regions at the given acoustic radiation force. The displacements were reduced by decreasing the applied acoustic pressure. These results clearly showed that the ARFI imaging system can be used to discriminate the relative stiffness of a target. As shown in Fig. 2a and b, we observed a distinct difference between the displacements in both the phantoms at 1.95 MPa and thereby used negative pressures of 1.95 MPa with excitation conditions corresponding to a spatial peak temporal average intensity (I_SPTA_) of 450.41 mW/cm^2^ for the subsequent experiments. Note that I_SPTA_ was the acceptable acoustic intensity within the limits regulated by the Food and Drug Administration (FDA). Furthermore, the displacements of five types of gelatin phantoms with different mechanical properties corresponding to the YM of each T-stage of cancer were measured at the determined excitation conditions. The mean displacements in the phantom with YM values of 0.94 (Normal), 2.81(T1 stage) and 3.49 kPa (T2 stage) according to the applied ARFI were measured to be 3.9 ± 0.63, 2.51 ± 0.5, and 2.18 ± 0.44 μm, respectively whereas the mean measured displacements in the phantom with a YM value of 8.89 (T3 stage) and 13.8 kPa (T4 stage) according to the applied ARFI were 1.39 ± 0.17 and 0.86 ± 0.17 μm, respectively. It was also found that the mean displacements of the phantom were significantly different [p-value = 0.43 × 10^−3^ (Normal and T1), p-value = 3.86 × 10^−3^ (T1 and T2), p-value = 1.32 × 10^−3^ (T2 and T3) and p-value = 0.14 × 10^−3^ (T3 and T4)]. These results thus demonstrated that the ARFI imaging system allowed us to distinguish the gelatin phantoms given such YM values, suggesting that it is capable of differentiating tissues with such different YM values. All of the experiments here were iterated ten times.

B-mode and ARFI imaging of the identical tissue-mimicking phantom utilized for evaluation of the multispectral imaging modality in the system were also performed using the ultrasound biomicroscopic system. The transducer used here was scanned over the target along the red arrow line in the spectral classified image with a step size of 40 μm. The B-mode and ARFI images of the tissue-mimicking phantom were acquired using the B/D-scan method (Fig. 2d and e). In the B-mode image (field of view: 2.5 mm × 4 mm) shown in Fig. 2d, stiffer regions (right) and softer regions (left) cannot be distinguished. However, in the ARFI image (Fig. 2e), the mechanical properties of the stiffer and the softer regions in the phantom can be discriminated. Figure 2e illustrates an overlaid image of an ARFI on a B-mode image. The color-mapped image shows the peak displacements of the target due to acoustic radiation force at 1.95 MPa (pulse duration: 0.22 ms). The yellow and red regions indicate the soft regions, whereas the blue-colored regions indicate the stiff regions. Thus, it was found that the regions of the phantom on the right side were stiffer than those on the left side. Additionally, B-mode and ARFI imaging of the bilayer gelatin phantoms in the axial direction was performed. As in the results above, in the B-mode image (Fig. 2g), stiffer regions and softer regions cannot be distinguished. However, in the ARFI images (Fig. 2h), the upper layer was softer than the lower layer in the phantom. Furthermore, the lateral and axial resolutions of ARFI imaging were measured with the bilayer phantoms in the lateral and axial directions using a modeling method which offers a point-spread function (PSF) from the first derivative of the edge-spread function (ESF) at the boundary of the ARFI images, as described previously^30^. The measured lateral and axial resolutions were 240 ± 10 μm and 188.3 ± 10 μm, respectively. Altogether, these results demonstrate that the multimodal biomicroscopic system is capable of performing multispectral, high-frequency B-mode and ARFI imaging of the same target simultaneously, suggesting that we can detect and delineate a target on the surface in the transverse direction as well as obtain structural and mechanical information about the target in the longitudinal direction using the system.

### Multimodal imaging of colorectal tumors *ex vivo*

**Figure 3:**
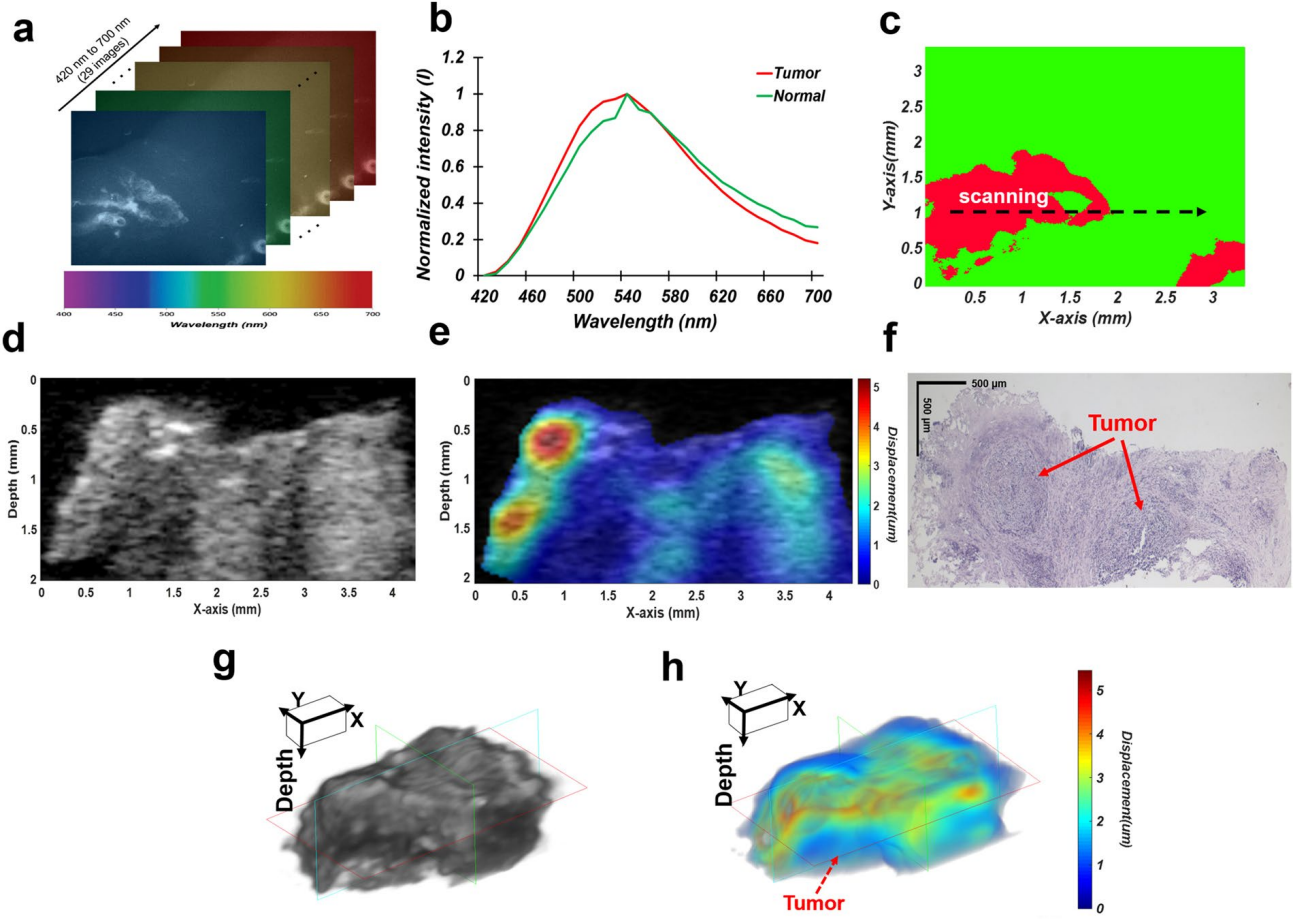
Multimodal imaging of colorectal tumors *ex vivo* using the proposed system. (**a**) Multispectral images obtained at the indicated wavelengths. (**b**) Normalized spectral signatures of colorectal tumors and normal colon tissues. (**c**) Multispectral classified image. The black dotted arrow indicates the scanning line. (**d**) A high-frequency B-mode image. (**e**) A high-frequency ARFI image overlaid with the corresponding B-mode image. (**f**) H&E stained image corresponding to the B-mode and ARFI image. The red arrows indicate tumor regions. (**g**) Three-dimensional B-mode and (**f**) ARFI image (2 mm × 2.8 mm × 4 mm in x, y, and depth). The pseudo-colored map indicates tissue displacements.

After the evaluation of our multimodal biomicroscopic system, extensive multimodal imaging of colon tissues including tumors excised from six cancer patients was performed. Figure 3 illustrates representative multimodal images (Supplementary Figure 1 describes other representative multimodal images and a H&E stained image corresponding to a multispectral classified image). Multispectral imaging and analysis of the regions of interest were initially carried out. Figure 3a and b show multispectral images within the wavelength range of 420 nm to 700 nm with a step size and a bandwidth of 10 nm as well as the normalized spectral signatures of colorectal tumors and normal colon tissues. The normalized spectral signatures of the normal colon tissues and colorectal tumors exhibited similar peaks around 550 nm. However, the spectral signatures for the colorectal tumors were broader than those for the normal colon tissues. Moreover, the intensity of the former was approximately 700 nm lower than that of normal tissues. Colorectal tumor regions on the surfaces of the colon tissues were clearly distinguished from normal tissues by spectral classification with these spectral signatures normalized as a reference, as shown in the spectral classified image (Fig. 3c).

In addition, B-mode and ARFI imaging of colon tissues was carried out along the black-arrow line on the spectral classified image to detect tumors in the longitudinal direction so as to discriminate between colorectal tumors and normal regions with high accuracy. Figure 3d and e indicate high-resolution B-mode and ARFI images of the excised colon tissues including tumors. In the B-mode image, tumor regions exhibit slightly darker intensity levels than the normal colon regions, but they are not clearly discriminated. In contrast, in the ARFI overlaid on a B-mode image, the colorectal tumors, shown in blue, were identified from the normal colon tissues. Typically, tumor regions were stiffer than normal regions. Finally, we confirmed our results by examinations of hematoxylin and eosin (H&E) stained tissue sections in the longitudinal direction corresponding to the B-mode and ARFI images. As shown in Fig. 3f, the regions at the pointed by the red arrows in the H&E stained image where were found to be stiffer in the ARFI image were indeed the tumor regions whereas the regions at the other part of the H&E stained image were the normal regions. The total acquisition time for ARFI, B-mode, and 29 multispectral images was 3 minutes 15 seconds. Note that the total time for examinations of excised tumors using our proposed system is still much shorter than that of examinations of human colorectal excised tumors using histopathological methods (preparation time: typically, over ~24 hours with the fixation of tissues). Therefore, these results suggested that the system has the potentials for rapid tumor characterizations compared to the histopathological methods. Furthermore, three-dimensional B-mode (Fig. 3g) and ARFI imaging (Fig. 3f) of the tumor was conducted using our system in order to investigate the potential for tumor detection. With 3D multimodal imaging using our system, the excised colon tissues including tumors and normal tissues could be examined with highly resolved anatomical, mechanical, chemical information pertaining to the tumors are located from the surface to deep inside the tissue in three dimensions.

**Figure 4:**
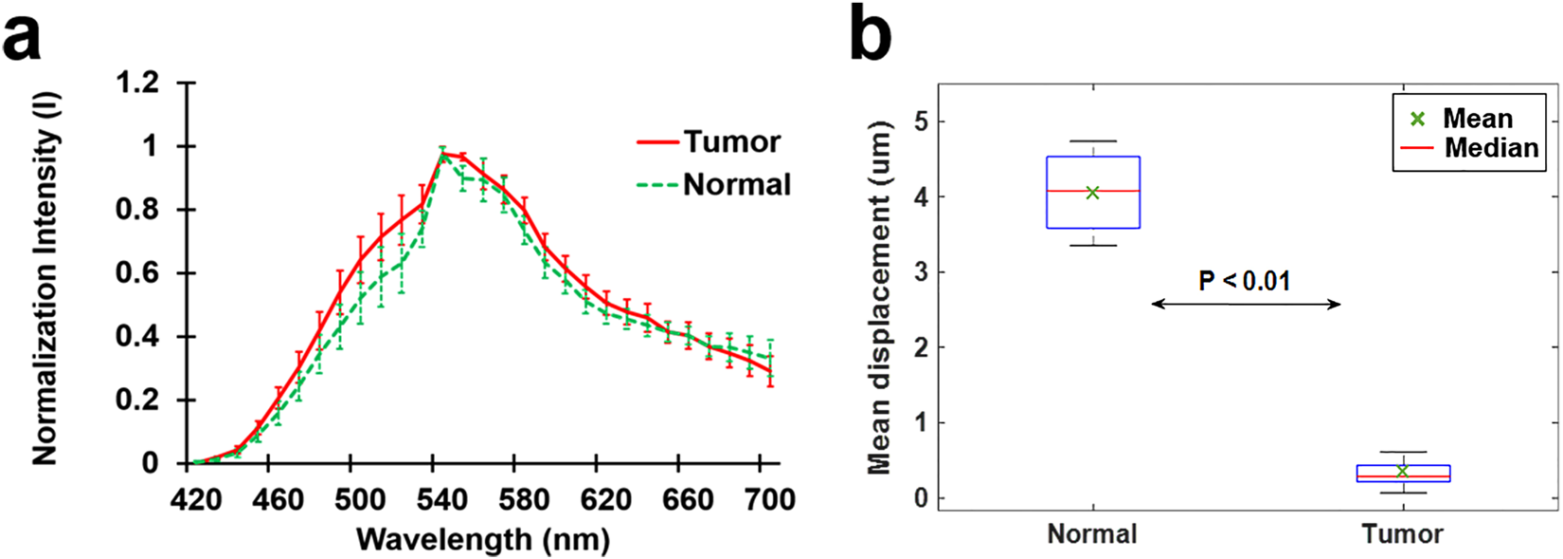
Statistical analysis of reference spectral signatures for multispectral imaging and mean displacements of tumors and normal regions induced by acoustic radiation force. (**a**) Normalized mean spectrum with the standard deviation (sample number = 6). (**b**) Box and whisker plot of the displacements of ARFI images (sample number = 6).

Finally, statistical analysis of reference spectral signatures for multispectral imaging and mean displacement of the tumors and normal tissues induced by acoustic radiation force were carried out. Figure 4a presents the normalized mean spectrum with the standard deviation of six samples of tumors and normal tissues. It was observed that the spectral signature of the tumors was distinguished from that of the normal colon tissues. The spectral signature of the tumors clearly exhibited higher intensity than that of the normal colon tissues at 520 nm. On the other hand, Fig. 4b illustrates the mean displacements of the ARFI images from the six samples. It was found in this case that the mean displacements between the normal tissues and tumors were significantly different (p-value = 0.41 × 10^−6^ < 0.01). These results thus demonstrated that the proposed system has the potential to discriminate between tumors and normal regions in excised human colorectal tissue samples.

## Discussion

In this study, it was found that a combination of optical and ultrasound imaging techniques offered biochemical, mechanical, and anatomical information about the colon tissues including tumors excised from cancer patients, suggesting that our results are more clinically applicable and meaningful as compared to tumors excised from animal models. In particular, the imaging modalities used in the system are complementary and synergetic with regard to the detection of tumors from the surface to deep inside tissues for the following reasons: (1) multispectral imaging allowed us accurately to detect tumor regions on the surface of tissues through a comparison of the spectral signatures of tumors and normal tissues, which may not be accomplished by ultrasound imaging; (2) high-frequency B-mode imaging provided anatomical information about the tissues deep inside tissues in the longitudinal direction at a high resolution; (3) ARFI imaging was capable of measuring the mechanical properties of tissues, thus allowing us to discriminate between mechanically different regions within tissues even with similar acoustic properties. Thus, due to the simultaneous use of multispectral, high-frequency B-mode, and ARFI imaging, we could detect tumors on the surfaces of excised tissues in the transverse direction as well as the locations and invasive depths of the tumors in the longitudinal direction.

Recently, many multimodal imaging systems have been developed via a combination of different imaging modalities. A multimodal imaging system including high-frequency B-mode, photoacoustic, and fluorescence life time imaging modalites^31^ was built to detect oral cancer whereas another type of a multimodal imaging system including ultrasound, photoacoustic, and fluorescence imaging modalities *in vivo* was built for real-time sentinel lymph node biopsy guidance^32^. Both the systems offered anatomical (internal structures such as soft tissues and vessels) and biochemical information on target tissues, but not mechanical information, which can be one of key features in the detection of tumors. In our previous study, the potentials of multimodal imaging including high-frequency B-mode imaging, fluorescence intensity, and confocal/spectral imaging for evaluation of treatment efficacy on tumors with novel nanoparticles were shown. The study showed that the multimodal imaging also allowed to obtain anatomical and biochemical information on tumor regions or evaluation of treatment efficacy of nanoparticles^33^. Recently, a multifunctional ultrasound biomicroscopic system including B-mode and ARFI/Shear wave elasticity imaging modalities has been developed. It could offer anatomical and mechanical information on tissues such as chicken liver, not chemical information on the surface of the tissues^30^. In contrast, the multimodal biomicroscopic system shown here allowed to perform high-frequency B-mode, ARFI, and multispectral imaging, thus enabling to obtain anatomical, mechanical and chemical information on tumors from surface to deep inside the tumors in the axial (B-mode and ARFI) and transverse (multispectral) direction. Hence, our system has distinct advantages over other previous systems in the examination of excised tumors from surface to deep inside the tumors.

The multispectral imaging modality in the system was used to detect diseased lesions on the surfaces of colon tissues. For the selections of light wavelengths in the multispectral imaging modality, a liquid-crystal tunable filter (LCTF) was incorporated into the system. LCTFs have been utilized for multispectral imaging in various biomedical applications^34,35^. Such a filter can offer more versatile wavelength and bandwidth selections than narrow optical bandpass filters. However, they require the use of a highly sensitive CCD camera to obtain images at different wavelengths of a high quality for multispectral imaging and analysis due to their inherent poor light transmission capabilities. Therefore, we utilized a highly sensitive CCD with a feasible quantum efficiency rate (>70% from 480 to 700 nm)^36^. In Fig. 1d, the boundary of the stiff regions including the fluorescent microspheres was clearly delineated and the distributions of the fluorescent microspheres could be observed in detail. In addition, the autofluorescence multispectral imaging and analysis of the excised tissues allowed us to discriminate between tumor and normal regions as shown in the previous studies^14–16^. Previous work showed that spectral imaging and analysis with the Mahalanobis distance algorithm offers sensitivity and specificity of 91% and 86% during the tumor detection of tissues *ex vivo*, respectively^37,38^. In contrast, it was found here that the sensitivity and specificity of spectral imaging and analysis were correspondingly ~92% and ~81% in the tumor detection of tissues *ex vivo*. In our results, the sensitivity of spectral imaging and analysis was somewhat lower than in previous results. This may have stemmed from the use of the different spectral analysis method, in which a spectral angle measure method was utilized^38^, as compared to that in the previous work, or it may have been due to use of excised tumors from different human. Thus, the results suggested that the proposed multispectral imaging and analysis technique in the system could accurately distinguish between the tumor and normal regions.

For high-resolution B-mode and ARFI imaging, we utilized a 45 MHz high-frequency ultrasound transducer with a low f-number of 1.5 as it can offer higher resolutions of less than 100 μm, appropriate for the imaging of the morphological structures and properties of excised tissues in detail^20^. In previous works, because of the normal colonic wall thickness ranged from 2 to 6 mm, endoscopic ultrasound imaging systems utilized high-frequency ultrasound transducers operating at over 20 MHz to image the detailed structure of the colorectal wall at 6 mm^39^. Also, when the depth of submucosal invasion by tumors is >1 mm as revealed upon an examination of an endoscopically resected tumor, additional surgical resection with dissection is recommended^40^. Thus, the examination of colorectal tumors with depths of 2–3 mm using high-frequency ultrasound imaging at 45 MHz would be sufficient to determine whether further tissues should be dissected. Moreover, for ARFI imaging, intense acoustic radiation force should be applied to tissues in order to induce displacements in them. Therefore, a high-frequency ultrasound transducer with a relatively low f-number of 1.5 was utilized to achieve intense acoustic radiation force and to realize a better spatial resolution at the point of focus. It was also observed here that the transducer generated negative pressure of 1.95 MPa at the point of focus when 65 V_pp_ was input. Due to the ARFI application at 65 Vpp, it was found that the relatively constant displacements in the tissue-mimicking phantom were induced at depths of ~2.5 mm. Note that the imaging depth can be extended by 4–6 mm as the input voltage to the transducer for the generation of acoustic radiation force for pushing the target, the input voltage to the transducer for the generation of a pulse, and the gain of the pulse receiver for the tracking beams are all increased. However, due to the use of the transducer with such a low f-number, resolution degradation of B-mode images at defocused regions outside of the depth of focus regions occurred. To complement the property of the low f-number transducer, multifocal scanning was conducted by moving the transducer in the longitudinal direction, with the images obtained at different focuses then combined after cropping of the focal regions in the images^41^.

The ARFI imaging technique was utilized here to measure the relative mechanical properties of excised tumors in the multimodal biomicroscopic system. The sensitivity levels of ARFI imaging when used to discriminate between normal and tumor regions here were found to be dependent on the cut-off mean displacements. Therefore, the sensitivity could not be definitely determined. As an alternative means of measuring the mechanical properties of excised tumors, a shear-wave-based high-frequency ultrasound imaging technique can be applied to the multimodal biomicroscopic system, as it may offer absolute mechanical information about target tissues. However, such a system also has a few shortcomings. For example, its spatial resolution is relatively low due to the boundary conditions of the samples compared to those associated with ARFI imaging techniques. It also requires two ultrasound transducers for excitation and tracking of the shear wave at a specific location^30^. Due to these reasons, we utilized the ARFI imaging technique for accurate detections of tumor regions within small colon tissue samples with high spatial resolutions. With measurements of the displacements of excised tissues with the ARFI application, the mechanical properties of the tissues could therefore be measured qualitatively via ARFI imaging, thus enabling us to discriminate between normal tissues and tumors, which is not always possible with B-mode imaging.

In the multimodal system, the optical and ultrasonic parts were located on opposite sides. This configuration may not be optimal for *in vivo* applications. However, the tumor is typically resected along with normal tissue at the margins of tumors for complete resection in cases where tumors form on the colonic wall. Therefore, it may be suitable to examine excised tissues *ex vivo* in such cases. For *in vivo* applications of the multimodal system, an endoscopic system with different configurations of the optical and ultrasonic parts should be redesigned. To do this, both of the probes in multispectral and high-frequency ultrasound imaging must face the same direction. This can be realized by a side-by-side arrangement of the probes or by the placement of the probe for spectral imaging in the middle of a ring-type high-frequency ultrasound transducer. This will remain as our future works for *in vivo* applications of the system.

Figure 3f illustrates an H&E stained image of a colon tissue sample in the longitudinal direction, which corresponds to the ARFI and B-mode images but not a multispectral classified image. In pathological examinations, only one-directional slide is allowed due to the protocol of sample preparation for H&E staining in paraffin blocks. Due to this limitation in the preparation of the H&E slice of a specimen, only one imaging direction can be chosen per sample for H&E staining. Thus, we here only included the H&E stained image in the axial direction, which corresponds to the B-mode and ARFI images. In previous work, tumor regions typically were stiffer than normal regions^42^. In the ARFI image (Fig. 3e), the deep blue-colored regions exhibited stiffer than the red-colored regions. The stiffness of a tissue is usually determined by the distribution of surrounding extracellular matrix (ECM) such as collagen^43^. In case of cancerous tissues, the ECM is also known as an origin of their enhanced stiffness^44^. As shown in the H&E image (Fig. 3f), tumor regions, which were indicated by arrows, exhibited dense ECM as well as the regions were well-matched with the darker and stiffer regions in the B-mode and ARFI images, respectively. Therefore, these results suggested that the B-mode and ARFI imaging allowed to determine whether tumors are localized in deep tissues without the laborious works needed for histopathological examination, although this method has inherent limitations such as low resolution compared to the histopathological examination. However, the results still showed slight misalignments between the ARFI and H&E images. Previous study reported that the volume of tissues commonly shrank by 33% when the tissue was embedded in paraffin wax for tissue sectioning, and moreover secondary shrinkage might be occurred by prolonged fixation in formalin^45^. Also, during the preparation of formalin-fixed paraffin-embedded tissue sections for histological analysis, the contour of a tissue section could be deformed from the original shape of a total mass due to the loss of soft tissues and a tilt in cutting edge in addition to the tissue shrinkage and distortion. Therefore, the misalignment may result from the loss of soft tissues, tissue shrinkage, and distortion occurred during the preparation of formalin-fixed paraffin-embedded tissue sections.

## Conclusions

In this paper, we built a multimodal biomicroscopic system based on high-frequency B-mode, ARFI and multispectral imaging techniques for rapid characterizations of excised tumors. The system offers highly resolved different/complementary information (molecular, anatomical, and mechanical information) about the excised tissues, thus allowing us to discriminate between tumors and normal tissues from the tissue surfaces to deep inside the tissue with high accuracy levels. These results thereby demonstrate that the multimodal biomicroscopic system proposed here has the potential to rapidly characterize excised human tissues qualitatively by means of a combination of high-frequency ultrasound and optical imaging modalities compared to the conventional histopathological examination. Thus, this system may become a very advantageous biomedical tool for use during surgical operations.

## Methods and Materials

### Multimodal biomicroscopic system based on high-frequency ultrasound and optical imaging techniques

**Figure 5:**
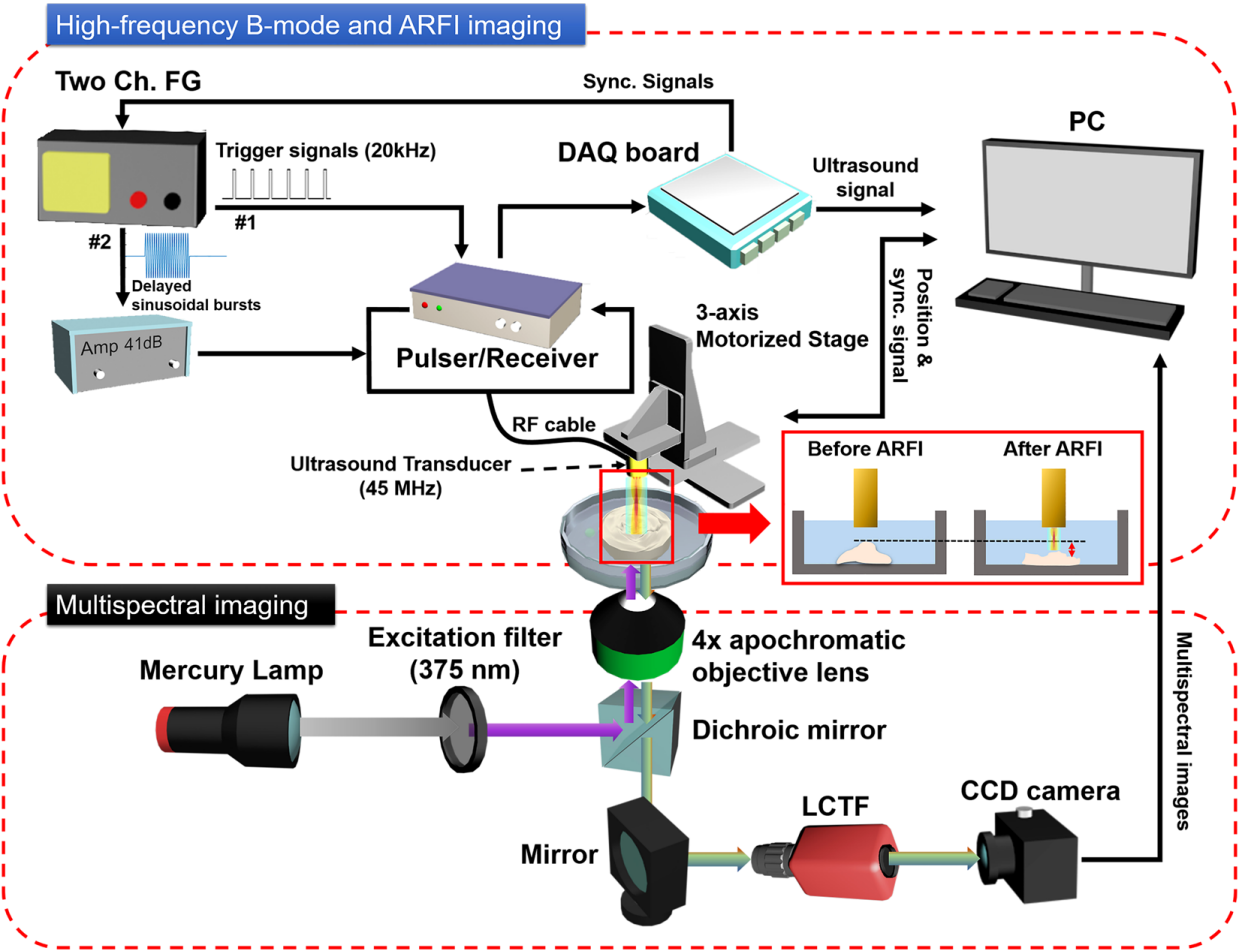
Block diagram of a multimodal biomicroscopic system.

A multimodal biomicroscopic system based on high-frequency ultrasound and optical imaging techniques was built. It included high-frequency ultrasound B-mode, ARFI and multispectral imaging modalities. The system consisted of two parts for high-frequency B-mode/ARFI and multispectral imaging, as shown in Fig. 5.

**Figure 6:**
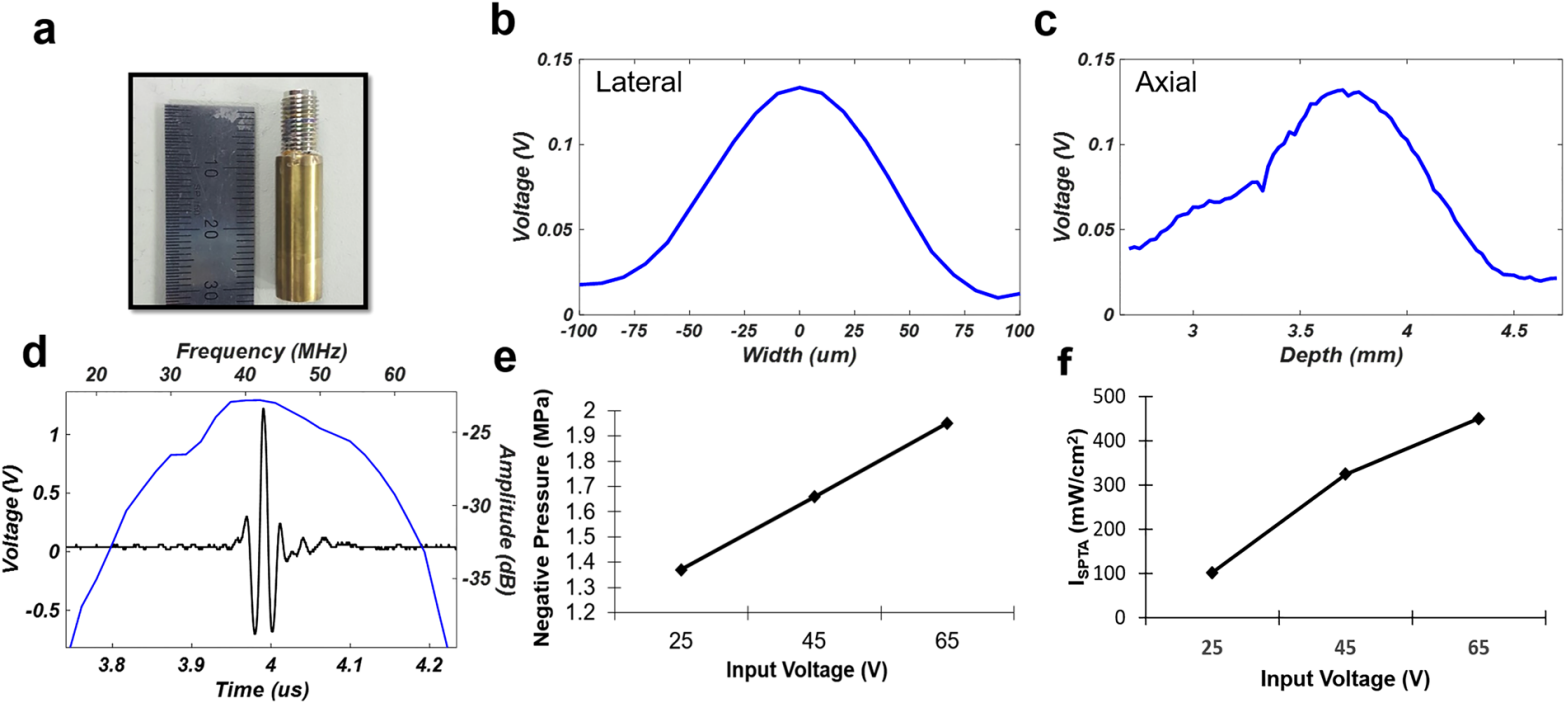
Characteristics of a 45 MHz lithium-niobate ultrasound transducer at focus. (**a**) Photographic image of a transducer. (**b**,**c**) Lateral and axial beam profile of a  transducer. (**d**) Pulse-echo characteristics of a transducer. (**e**) Negative pressure versus the amplified input voltage to a transducer. (**f**) I_SPTA_ versus amplified input voltage to a transducer.

For high-frequency B-mode and ARFI imaging, a 45 MHz lithium-niobate ultrasound transducer was fabricated and attached to x-, y-, and z-axis motorized stages, which allows both two- and three-dimensional ultrasound imaging. Figure 6a presents a photographic image of a single-element lithium-niobate ultrasound transducer. The center frequency of the transducer was 45 MHz, and its −6 dB bandwidth was 58%, as shown in Fig. 6d. The aperture diameter and focal length of the transducer were 2.5 mm and 3.75 mm, respectively (f-number: 1.5). The −6 dB lateral and axial beam widths were measured to be 80 μm and 600 μm by quantification of the beam profiles at a focus level obtained (Fig. 6b,c). In addition, the negative pressures versus the input voltages [Frequency: 45 MHz, number of cycles: 10,000] were quantified by using acoustic intensity measurement system (AIMS, ONDA). The negative pressures were 1.37, 1.66, and 1.95 MPa and the corresponding I_SPTA_ values were 101.6, 324.6 and 450.41 mW/cm^2^ when the amplified input voltages to the transducer were 25, 45, and 65 V_pp_, respectively, as shown in Fig. 6e and f. In the acoustic intensity measurement system, a hydrophone (HGL-0085, ONDA) was utilized to measure the beam profiles, negative pressures, and I_SPTA_ values. The diameter of the hydrophone was 85 μm.

The transducer was driven by a pulser-receiver (DPR500, JSR Electronics) to perform high-frequency B-mode and ARFI imaging simultaneously. After the transducer was activated by a pulse from the pulser-receiver, echo signals from the target were detected by the transducer and then amplified by 26 dB in the pulser-receiver. Subsequently, they were transferred to an analog-to-digital converter (CS122G1, Gage), which offered a 12-bit quantization resolution and a 250 MHz sampling frequency, and then  recorded in a custom-built program in order to obtain B-mode and ARFI images. For ARFI imaging, 12 trigger signals from the first channel of a function generator (AFG3252, Tektronix) (FG#1) were input to the pulser-receiver with a pulse repetition frequency (PRF) of 20 kHz to acquire reference and tracking signals for the measurement of displacements of the target due to the acoustic radiation force applied to the target. In addition, the acquisition frequency per each scan line for ARFI imaging was 100 Hz. 45 MHz sinusoidal bursts (number of cycles: 10,000) from the second channel of the function generator (FG#2) amplified by a custom-built power amplifier (gain: 41 dB) were input to the transducer to generate acoustic radiation force. Here, the sinusoidal bursts were input to the transducer after acquisition of a reference signal. For the synchronization of all of the equipment utilized for ARFI imaging, synchronization signals from an analog-to-digital converter were input to both the pulser-receiver and the function generator.

For multispectral imaging in the system, a LCTF (KURIOS-VB1, Thorlabs) allowing wavelength selections of light with a bandwidth of ~10 nm was incorporated into a conventional inverted microscope (IX73, Olympus). Illumination light from a mercury lamp passed through an excitation filter (ex: 375 nm) and was directed to a target of interest by a dichroic mirror (reflection band: ~370–410 nm) and an apochromatic 4x objective lens for excitation of the target. The autofluorescence light emitted from the target was collected by the objective lens and filtered by the LCTF, after which it was recorded using a highly sensitive CCD camera (Pco. Edge 4.2, PCO). The spatial and spectral resolutions of the multispectral imaging system were 13 μm × 13 μm and 10 nm, respectively. The field of view of the image was 3.3 mm × 3.3 mm.

### Construction of a tissue-mimicking phantom and preparation of a colorectal tumor for a system evaluation

For an evaluation of the performance of the multimodal biomicroscopic system developed here, a total of nine tissue-mimicking phantoms with different gelatin gel concentrations were fabricated in order to mimic the YM (Tumors: 13.8, 8.89, 7.51, 3.49, and 2.81 ± 0.5 kPa, Normal: 0.94 ± 0.5 kPa) corresponding to the T-stage of colorectal tumors and normal tissues^46^, as described previously^47^. Note that the stages of colorectal cancer follow the guideline of the American Joint Committee on Cancer Staging (AJCC). The cancer staging is divided into T stages according to the depth of invasion into the large intestine. The N stage is divided according to extent of lymph node invasion, and M stage is evaluated by distant metastasis. During the construction of the tissue-mimicking phantoms, gelatin gels were hydrated with deionized water (100 g) and an n-propanol (8.9 g) solution, respectively, to ensure that the sound speed of the phantom was similar to that of the tissue studied here. The mixture was then placed into a vacuum oven to remove residual air bubbles. Graphite powders (5.5 g) were then added to the mixture to enhance the echogenicity of the phantom. Formaldehyde (0.5 g) was also used to raise the melting point by increasing the cross-linking of the gelatin gels. After pouring the mixture into a petri dish, it was quickly placed in an ice bath in order to prevent precipitation of the graphite powders, thus realizing a homogenous phantom. The region of the gelatin gels with a higher density level, which contained fluorescent microspheres (0.01 g), was constructed in the remaining half of the petri dish after the region of the gels with a lower density level was constructed. Note that the highly concentrated regions of the gelatin gels denote stiffer regions than the regions with lower density levels. The size of the fluorescent microspheres used was 15 μm. They emit fluorescent light at 415 nm when they are excited by light at a wavelength of 375 nm.

Frozen tissues of patients with colorectal tumors were provided by Keimyung Human Biobank of Keimyung University Dongsan Medical Center. The use of the patient tissues was approved by the Institutional Review Boards (IRBs) of Keimyung University Dongsan Medical Center and Daegu Gyeongbuk Institute of Science & Technology. In addition, all experiments were performed in accordance with the relevant guidelines and regulations and informed consent was obtained from participant. The frozen tissues were slowly thawed and then were immersed in Hank’s balanced salt solution (HBSS) after washing the tissues with fresh HBSS solution several times to perform multimodal imaging for tumor characterization using our system, as described previously^48^. Note that the multimodal imaging system can be also applied to perform multimodal imaging system of fresh tumor characterizations.

Immediately after the imaging analysis, they were fixed in 4% paraformaldehyde for 24 hours and embedded in paraffin. Tissue sections 4 μm thick were stained with hematoxylin for 10 minutes, washed, and then stained with eosin for 2 minutes. After washing with water, the slides were gradually dehydrated in 50%, 70%, 90%, and 100% ethanol. Histological images were captured with a light microscope (Leica ICC50 HD, Leica Microsystems, Wetzlar, Germany).

### Multimodal imaging and analysis of the phantoms and colorectal tumors

For the multispectral imaging and analysis, the target was excited by light at 375 nm and images at consecutive wavelengths from 420 nm to 700 nm were acquired with a step size of ~10 nm and a bandwidth of ~10 nm. Spectral classification of the images was then performed using a program developed previously with predefined reference signatures^37^. The reference signatures were acquired from the regions of the gelatin gels with higher and lower density levels as well as from the normal tissue and the tumor regions in the multispectral images.

During the determination of the relative stiffness of a target at each scan line for the ARFI imaging of the targets, the first trigger signals were input to the pulser-receiver to obtain the reference signals from the target, followed by the application of acoustic radiation force to the target. Subsequently, the following trigger signals were input to the pulser-receiver to track the displacements of the target due to the applied acoustic force. The procedures described above were conducted at each scan line after the mechanical movement of the transducer to scan in a step-by-step manner. The step size for the linear scan was 40 μm, which was equal to half of the −6-dB lateral beam width. Here, the displacements of the target due to the acoustic radiation force were quantified by a conventional normalized cross-correlation method for ARFI imaging^49^. To do this, a reference, in this case the RF signals by averaging the three RF signals before the application of acoustic radiation force, as well as data from the tracking of RF signals were both up-sampled to 1 GHz using a cubic-splines method to preserve the original signals. The reference signal and tracking segmented signal with a length of 6 λ were cross-correlated to find the maximum correlation coefficient. The ARFI image was a pseudo-mapped image with peak displacements of the tissues induced by the acoustic radiation force. Thus, the regions shown in red in the ARFI images represent softer regions than those shown in blue. This procedure was carried out in the regions of interest within the depth of focus of the transducer. In addition, in order to compensate for the narrow depth of focus of the high-frequency ultrasound transducer, which however offers a better lateral resolution, a multi-depth scanning method known as the B/D-scan method was utilized^50^ while scanning the transducer with a step size of 300 μm, equivalent to half of the axial beam width, in the axial direction. This method can be used to ensure that the target is moved by the acoustic radiation force in the focal region of the transducer, ensuring that the homogeneity of the B-mode and ARFI image for each depth can be achieved. The peak displacement map was overlaid with a B-mode image created with the reference signal through a conventional B-mode image reconstruction method^27^.

### Statistical analysis of spectral signatures and mean displacements of colorectal tumors and normal tissues

For a statistical analysis, the mean and standard deviation of the spectral signatures and mean displacements of colorectal tumors and normal tissues were obtained with a sample size of n = 6. A two-tailed paired t-test was performed with the level of significance set as a p-value < 0.01.

## Electronic supplementary material

Supplementary Figure
