# Supplementary material for: A Multimodal Biomicroscopic System based on High-frequency Acoustic Radiation Force Impulse and Multispectral Imaging Techniques for Tumor Characterization Ex vivo
Source: Sci Rep. 2017 Dec 13;7:17518. doi: 10.1038/s41598-017-17367-1 (PMC5727531; doi:10.1038/s41598-017-17367-1)

Supplementary materials for

A Multimodal Biomicroscopic System based on High-frequency Acoustic Radiation Force Impulse and Multispectral Imaging Techniques for Tumor Characterization *Ex vivo*

Jihun Kim, Anna Seo, Jun-Young Kim, Sung Hyouk Choi, Hyung-Jin Yoon, Eunjoo Kim, and Jae Youn Hwang

Supplementary Figure. Multimodal images of colorectal tumors *ex vivo* using the proposed system

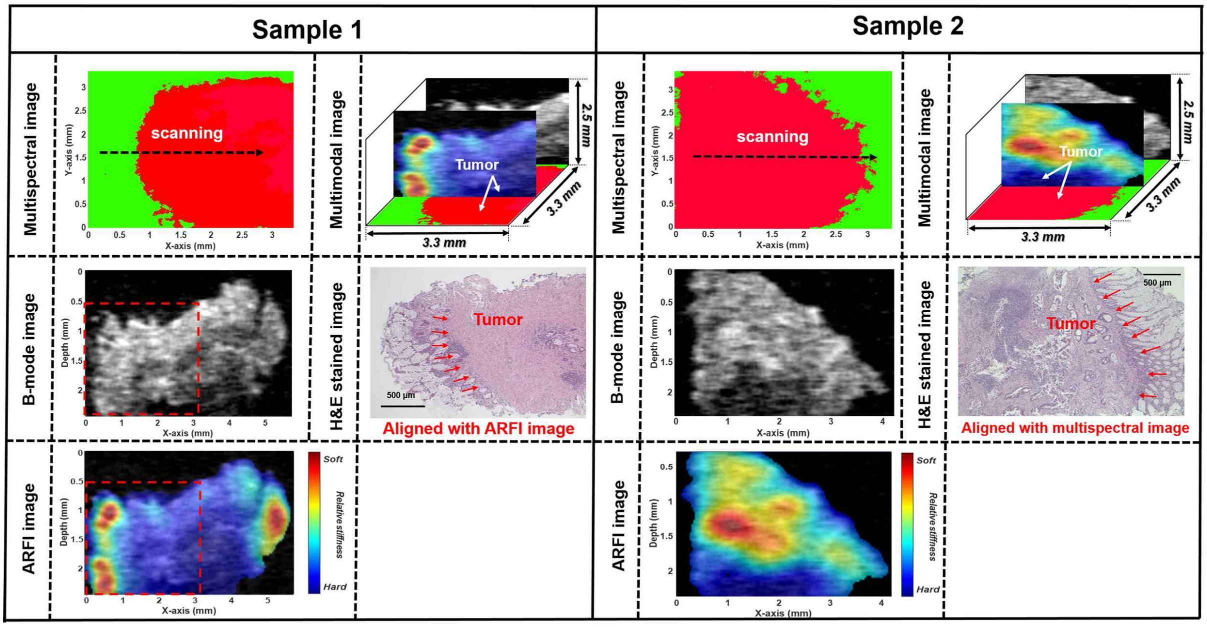

Supplement: Supplementary file 1 — Supplementary Figure [file 41598_2017_17367_MOESM1_ESM.pdf]
